# Blood biochemical parameters of Murrah buffalo (*Bubalus bubalis*) reared in the high salinity area of Bangladesh

**DOI:** 10.5455/javar.2022.i643

**Published:** 2022-12-31

**Authors:** Rukhsana Amin Runa, Md. Mazharul Islam, Moinul Hasan, Mst. Antora Akter

**Affiliations:** Department of Surgery and Obstetrics, Faculty of Veterinary Science, Bangladesh Agricultural University, Mymensingh 2202, Bangladesh.

**Keywords:** blood parameters, buffalos, saline water, skin lesions

## Abstract

**Objectives:**

The study’s goal was to determine the impacts of drinking saline water on the biochemical parameters of Murrah buffalo.

**Materials and Methods:**

Twenty Murrah buffalo of both sexes, ranging in age from 6 months to 11 years, were randomly selected. 10 ml of blood samples were collected from each buffalo and processed to separate serums. The supplied drinking water and feed samples were also collected. Blood parameters–glucose, alanine-aminotransferase (ALT), aspartate-aminotransferase (AST), creatinine, urea, uric acid, magnesium, phosphorous, calcium, zinc, potassium, sodium, and chloride–were analyzed. Different clinical manifestations of selected buffalo were also recorded.

**Results:**

The buffaloes with skin lesions had higher serum concentrations of AST, creatinine, zinc, sodium, and chloride compared to another group. Significant age effects were found for serum concentrations of glucose (*p* < 0.05), creatinine (*p* < 0.01), uric acid (*p* < 0.001), phosphorus (*p* < 0.05), and potassium (*p* < 0.05). No significant age and gender effects were found for AST, ALT, urea, magnesium, calcium, zinc, sodium, and chloride. The serum concentration of creatinine was higher in female buffaloes, whereas the uric acid concentration was lower in female buffaloes compared to males.

**Conclusion:**

The blood parameters that were measured and remained within reference ranges show that Murrah buffaloes can adapt to saline water with little to no impairment to their liver and renal functions. The differences in the ages and sexes of the animals within the study groups highlight that mature female buffaloes suffer more from skin disorders and attempt to adjust to exotic conditions by changing the functioning of their liver and kidney.

## Introduction

Providing enough high-quality drinking water for farm animals is a normal farm management concern that is frequently disturbed by a variety of environmental factors. Changes in water quality, such as the salinization of fresh-water sources caused by climate change [1], represent a serious threat to livestock populations’ health and production. Excess dietary salt consumption causes hypertension in animals [2,3], as well as increased pulse and respiration rates, water retention, muscle cramps, and neurological problems [4,5]. In sheep, increased water salinity reduced blood and plasma volume, extracellular and interstitial fluids, and urea concentrations, while milk production was decreased in cows [6,7,8]. When lambs were exposed to 1.5% saline water for 3 weeks, Yousfi and Ben Salem [9] observed increased plasma glucose, creatinine, urea, and gamma-glutamyl-transferase.

The salt concentration on the exterior of the cell membrane is higher than on the interior of the cell membrane when animals consume seawater. Osmosis is impeded by the salt content differential, which causes cell shrinkage [10]. The body increases urination to eliminate excess Na+ from extracellular fluids to return to an isotonic state, resulting in dehydration, muscle cramps, thirst, and a dry mouth and eyes [10]. The body attempts to compensate for the fluid loss by increasing heart rate and constricting blood arteries, resulting in a reduced blood supply to the brain and other organs, resulting in a coma and eventually death [10].

Assays for aspartate aminotransferase (AST) and alanine aminotransferase (ALT) are needed to figure out how well the liver is working. AST and ALT levels are notably high in individuals suffering from cirrhosis and liver injury [11]. Runa et al. [12] discovered that the AST and ALT values were slightly higher in goats with unrestricted access to drinking 1.2% salt water. When Barki sheep were given normal drinking water, moderately salty drinking water (0.5%), and very salty drinking water (0.9%), Ghanem et al. [13] found the same thing. Similarly, sheep and camels’ AST and ALT were significantly impacted by the salinity (1.35%–1.4%) of drinking water [6]. Instead, both enzymes remained unaltered even though the saline water exposure of goats gradually increased (between 0.25% and 1.5%) [14].

Creatinine is eliminated through glomerular filtration and is a dependable indicator of renal function [15]. Serum creatinine levels increased and remained over the upper limit of the reference range [16], when goats were given a higher concentration of salty water (1.2%) for a longer period of time, according to Runa et al. [12], suggesting that prolonged salty water consumption had a deleterious impact on renal function. Serum creatinine after saline water ingestion was highest in the freshwater phase (up to 2% salt) [17]. Higher salt concentrations were observed to considerably increase glomerular filtration rate (GFR) in the goats, indicating that the kidneys more effectively filter wastes such as urea, uric acid, and creatinine [7].

Bangladesh Milk Producer’s Co-operative Union Ltd. (Milkvita) has imported Murrah buffalo from the Haryana province of India for its Tekerhat, Madaripur, and Raipur, Lakshmipur stations to improve milk production in Bangladesh. The imported buffalo were tormented by severe skin disorders such as thickening and cracking of the skin, as well as skin erosion that went from leg to udder, during their early days of acclimating to the new climate. Following a clinical examination, it was primarily diagnosed that this could have been caused by higher salinity levels in drinking surface water. Drinking saline water consistently for the first time in their lives is thought to influence the physiological parameters of the imported Murrah buffalo. Although water salinity is not found in the Haryana province of India, where the Murrah buffaloes originate, it is a common problem in the areas covered by Milkvita’s Raipur station.

Understanding the physiological factors underlying buffaloes’ ability to acclimatize to a saline load is essential for establishing long-term strategies for producing buffaloes in areas where the water contains high salt concentrations. So far, several studies have been done on the impact of water salinity on human health [18–21]. However, there is a scarcity of information on the health effects of ruminants, notably buffalo, drinking saline water in coastal areas of Bangladesh. As a result, the study is unique in that it determines the effects of increased water salinity on buffalo health, which could aid in exploring appropriate adaptive physiological responses. The study’s objectives were to determine the effects of saline drinking water on buffalo blood electrolytes, liver, and kidney functions, as well as identify potential health risks for buffalo in salinity-affected areas of Bangladesh.

## Materials and Methods

### Ethical approval

Procedures performed in this study were approved by the Animal Welfare and Experimentation Ethics Committee (AWEEC) of Bangladesh Agricultural University, Mymensingh-2202 [AWEEC/BAU/2021(13)].

### Research area

The research was conducted at Milkvita Buffalo Farm in Raipur, Lakshmipur, situated at an altitude of 12 m above mean sea level, with latitude and longitude positions of 23°01ʹ59.88ʺ N 90°46ʹ30.00ʺ E, respectively. 

### Animals

Twenty Murrah buffalo of both sexes, with or without evidence of clinical symptoms, were randomly selected. All buffalos had an average body weight of 295 kg and their age ranged from 6 months to 11 years. Buffalos were classified as “young” (number of animals = 10) or “adult” (number of animals = 10) when younger or older than 2 years, respectively.

### Collection and processing of blood

After sterilizing the area to be punctured, blood (10 ml/animal) was drawn through the jugular vein puncture, causing the least disruption to the animal during collection. Samples were transferred into serum tubes (4 ml Clot Activator, Cure, Shengbang Laboratory, China) for collecting serum. All samples were immediately placed in the ice box containing ice bags for cooling the samples and transported to the laboratory for further processing. Then the blood samples were centrifuged by a centrifugal machine (DSC-200-A2, Table Top Centrifuge, Digisystem Laboratory Instruments Inc., Taiwan) at 3,000 rpm for 15 min at room temperature. The serum samples were separated and pipetted into labeled glass vials and stored at −20℃ until further analysis.

### Laboratory analysis of samples

Serum samples were transferred to the Mohammad Hossain Central Laboratory, Bangladesh Agricultural University to measure blood electrolytes and other biochemical parameters: potassium, calcium, zinc, phosphorous, chlorine, sodium, magnesium, glucose, urea, uric acid, creatinine, ALT, and AST. ALT, AST, glucose, urea, and uric acid were determined by a UV spectrometer (T80, USA) using Reactivos GPL (Barcelona, Spain). Phosphorous and creatinine were measured by UV spectrometer (T80, USA) using Vitro Scient (Egypt) and Linear (Barcelona, Spain), respectively. Zinc and magnesium were also measured using Chema Diagnostica (Italy).

### Water and feed sample analysis

Drinking water samples from ponds and tube wells and supplied feed (grass and concentrates) samples, were randomly collected from the study areas. A sodium chloride refractometer (HI96822 Seawater Refractometer, HANNA Instruments) was used to measure the salt (NaCl) concentration of collected drinking water. The pH of supplied water samples was also measured by a pH meter (HI2211 pH/ORP Meter, HANNA Instruments). The feed sample was stored for further analysis for dry matter intake and other mineral contents of the feed. Water and feed samples were transferred to the Department of Agricultural Chemistry at the Bangladesh Agricultural University to analyze the mineral contents of drinking water and feed. In addition, feed samples were also taken to the Interdisciplinary Institute of Food Security at the Bangladesh Agricultural University for analysis of crude protein, fiber, crude fat, ash, moisture, and carbohydrate. All values for feed and water are summarized in Tables 1 and 2. According to Runa et al. [12], methods were used to determine the percentage of crude protein, ash, crude fat, fiber, sulfur, phosphorous, sodium, potassium, calcium, magnesium, and bicarbonate in the feed sample. 

### Recording of clinical data

Also, information about how the buffalo at the farm in the project area looked when they got sick was written down to see if there was a link between getting sick and drinking salty water.

**Table 1.** Chemical composition of supplied feed samples of buffalo.

Compositions	Amount
Crude protein (%)	2.59
Fiber (%)	44.71
Crude fat (%)	3.25
Ash (%)	11.41
Moisture%	15.03
Carbohydrate (%)	23.01
Ca (%)	1.603
Mg (%)	0.656
P (%)	0.581
Na (mg/l)	25.925
K (mg/l)	50.583

**Table 2.** Mineral components of supplied drinking and bathing water of buffalo.

Components	Drinking water	Bathing water
Ca (mg/l)	184.368	27.254
Mg (mg/l)	30.138	17.499
P (mg/l)	0.389	2.771
S (mg/l)	1.137	4.014
HCO^3^- (mg/l)	558.8	122.0
Na (mg/l)	166.112	23.489
K (mg/l)	120.342	128.761
Cl (mg/l)	225.930	39.988

### Statistical Analysis

All data were presented as mean ± SEM. To determine the significant difference between the two groups in each parameter, an independent samples *t*-test (unpaired and parametric) was performed. The data obtained in the present investigation were analyzed with Statistical Package for the Social Sciences software. Probability *p* < 0.01 and probability *p* < 0.05 were considered statistically significant.

## Results and Discussion

In order to study the impacts of water salinity on the biochemical parameters of buffalo, Table 3 shows the concentration of salt (NaCl) and pH of the provided water sample. On the pH scale, the pH of rivers and lakes typically ranges from 5 (acidic) to 9 (basic), whereas the average pH of ocean water is closer to 8.2 (slightly basic). It varies, nevertheless, according to the location, time of day, and depth of the water sources [22]. Due to the effects of the carbonate-buffer system, the pH of the majority of the water may be maintained to some degree. The supplied drinking water had a sodium chloride content of 2 ppt, which is considered moderately saline water [23]. It was 4–5 times higher than the upper limit of salt concentration recommended for fresh drinking water (0–0.5 ppt). Animals may take up to 1% (10 ppt) of salt in their drinking water without harming their health, according to several studies [14,24]. So, it is possible that the drinking water provided may affect the physiological characteristics of the Murrah buffalo’s adaptation in the study area.

**Table 3.** Salt concentration and pH of supplied water sample.

Supplied water sample	Salt (NaCl) concentration (ppt)	pH
Drinking water	2	6.95
Bathing water	0	6.60

The findings for all blood metabolites dependent on the presence or absence of skin lesions are presented in Table 4. The concentration of uric acid in the blood was shown to have the only meaningful influence. Compared to other buffaloes, those with skin lesions showed greater serum concentrations of AST, creatinine, zinc, sodium, and chloride.

Zinc is required to regulate several enzymes, immunological responses, neurological processes, and protein synthesis [25]. A deficiency of zinc results in eye and skin lesions, hair loss, immunological dysfunction, and altered endocrine functioning, whereas an excessive amount of zinc causes nausea, vomiting, and headaches as the signs of its toxicity [26]. Therefore, adjusting zinc levels to their normal range is important for sustaining cellular functions and biochemical reactions in the body. Zinc transporters transport zinc between extracellular and intracellular compartments and help maintain serum zinc concentrations [27]. Impairment of zinc and zinc transporter function is strongly associated with clinical diseases of various organs in the body [27].

Sodium and potassium levels in the animal body are influenced by dietary zinc, and supplementing with zinc seems to improve kidney salt balance [28,29]. Recent hypertension research has linked low Zn levels to a Na+-Cl co-transporter (NCC)-dependent mechanism that causes renal Na+ retention at the distal convoluted tubule [30]. Kumar [31] found that Zn indirectly affects sodium levels and blood pressure by influencing a wide range of proteins that support the NCC’s function. Furthermore, if consumed sodium is not properly regulated by the NCC-dependent mechanism, individuals’ health can suffer, regardless of whether they consume too much or too little sodium. In our study, we found that all of the study animals had serum zinc levels many times higher than the reference ranges. This might be attributed to the impairment of the Zn regulation system in the body caused by higher amounts of Na+ ingestion through consistent consumption of salty water. The mechanism, however, is not clearly known.

Lower urea clearance in the urine as a result of severe renal impairment and a corresponding considerable drop-in GFR could explain the highest levels of urea identified in our study. Greater urea production could suggest that the feed is consuming a lot of protein, which is linked to increased urea synthesis and, as a result, higher blood urea levels [7]. Increased plasma or serum urea can be caused by increased urea production, decreased urea excretion, or a combination of the two. The differences in serum urea concentrations between young and older buffaloes in our investigation could be attributable to age [7].

The age-wise effect of drinking saline water on different blood parameters in Murrah buffalo is presented in Table 5. Significant age effects were found for serum ­concentrations of glucose (p < 0.05), creatinine (p < 0.01), uric acid (p < 0.001), phosphorus (p < 0.05), and ­potassium (p < 0.05). For AST, ALT, urea, magnesium, calcium, zinc, sodium, and chloride, no significant age effects were observed. All of the blood levels tested were within the normal range, except for AST, ALT, and zinc. In this study, serum AST and ATL values were lower; however, serum zinc concentrations were significantly higher than the standard range. Drinking water salinity (1.35%–1.45%) had a significant effect on serum ALT and AST in sheep and camels [6,32]. It suggests that increasing salt in drinking water causes hepatic hyperfunction or hypofunction in sheep, especially when nutritional intake is lowered. In this study, however, both enzymes (ALT and AST) were determined to be below the standard range. It is possible that the liver malfunctioned as a result of ingesting saline water. This is in line with the findings of Abdellah et al. [33], who reported that buffaloes’ hepatic enzyme levels dropped after drinking saline water.

**Table 4.** Comparison of different blood parameters based on the presence and absence of skin lesions in the Murrah buffalo (mean ± SEM).

Parameters	Skin Lesions		*p*- values	Reference Value
	Present	Absent		
Glucose (mg/dl)	61.51 ± 6.99	79.26 ± 8.74	0.22	22.33–97.49 [33]
AST (U/l)	10.40 ± 0.86	8.92 ± 0.65	0.21	24.21–93.40 [33]
ALT (U/l)	5.44 ± 0.63	6.08 ± 0.43	0.42	7.17–48.48 [33]
Creatinine (mg/dl)	2.35 ± 0.19	1.94 ± 0.24	0.31	1.07–2.52 [33]
Uric Acid (mg/dl)	2.27 ± 0.33	3.51 ± 0.26	0.02	
Urea (mg/dl)	45.06 ± 2.12	47.32 ± 2.09	0.53	
Magnesium (mEq/l)	2.17 ± 0.08	2.06 ± 0.03	0.13	2.20–3.93 [33]
Phosphorus (mg/dl)	2.87 ± 0.46	3.94 ± 0.57	0.26	5.71–10.35 [33]
Calcium (mg/dl)	8.36 ± 1.43	8.69 ± 0.50	0.78	8.72–12.3 [36]
Zinc (mg/dl)	124.48 ± 7.25	115.57 ± 3.81	0.25	0.05–0.13 [36]
Potassium (mmol/l)	4.96 ± 0.20	5.09 ± 0.09	0.48	3.55–5.9 [36]
Sodium (mmol/l)	158.78 ± 12.65	148.31 ± 5.79	0.40	125.7–164.8 [36]
Chloride (mmol/l)	114.00 ± 7.22	99.11 ± 7.14	0.23	73.41–116.78 [33]

SEM = standard Error of Mean

**Table 5.** Age-wise effect on different blood parameters of Murrah buffalo by drinking saline water (mean ± SEM).

Parameters	Young	Adult	*p*- values	Significance	Reference Value
Glucose (mg/dl)	87.10 ± 11.11	60.77 ± 4.85	0.043	*	22.33–97.49 [33]
AST (U/l)	8.57 ± 0.76	10.15 ± 0.70	0.144		24.21–93.40 [33]
ALT (U/l)	5.89 ± 0.34	5.89 ± 0.64	0.997		7.17–48.48 [33]
Creatinine (mg/dl)	1.57 ± 0.17	2.56 ± 0.24	0.003	**	1.07–2.52 [33]
Uric Acid (mg/dl)	4.09 ± 0.09	2.19 ± 0.20	0.000	***	
Urea (mg/dl)	48.79 ± 2.32	44.49 ± 2.04	0.181		
Magnesium (mEq/l)	2.04 ± 0.04	2.15 ± 0.05	0.095		2.20–3.93 [33]
Phosphorus (mg/dl)	4.57 ± 0.70	2.68 ± 0.30	0.023	*	5.71–10.359 [33]
Calcium (mg/dl)	8.78 ± 0.57	8.39 ± 0.92	0.723		8.72–12.3 [36]
Zinc (mg/dl)	115.45 ± 3.88	121.04 ± 5.81	0.434		0.05–0.13 [33]
Potassium (mmol/l)	5.23 ± 0.08	4.87 ± 0.12	0.023	*	3.55–5.9 [36]
Sodium (mmol/l)	150.78 ± 7.62	152.13 ± 8.26	0.906		125.7–164.8 [36]
Chloride (mmol/l)	106.21 ± 3.20	100.94 ± 10.91	0.649		73.41–116.78 [33]

* p < 0.05.

** p < 0.01.

*** p < 0.001.

SEM = Standard error of mean.

Table 6 shows the effect of various blood parameters on different sexes. Only the serum concentrations of creatinine (*p* < 0.01) and uric acid (*p* < 0.01) showed a significant sex influence. Female buffaloes showed higher levels of creatinine in their blood than male buffaloes but lower levels of uric acid. It is generally known that various gender-related factors contributed to males having higher serum uric acid levels than females. The protective effect that females seem to have may be linked to estrogen levels. It is well known that estrogen encourages the excretion of uric acid. Because estrogen is a uricosuric substance, in this case, a sex difference in the risk of hyperuricemia may exist [34]. An earlier investigation by Antón et al. [35] revealed that lower tubular urate post-secretory reabsorption and greater plasma estrogen levels in females were the causes of their higher renal clearance of urate [35]. In our study, it is yet unknown why there is a difference between males and females in the connection between high creatinine and uric acid. The creatinine level in adult female buffaloes was greater than the upper limit of the reference range [33], showing that drinking saline water regularly has some influence on renal function. In a similar investigation, Zoidis and Hodjigeorgiou [17] discovered that goats given up to 2% salt water had the highest plasma creatinine concentrations. This finding could be explained by changes in the GFR. Larger salt concentrations resulted in a higher filtration burden of waste products such as urea, uric acid, and creatinine in the kidneys, leading to a significantly higher GFR [7].

**Table 6.** Sex-wise effect on different blood parameters of Murrah buffalo by drinking saline water (mean ± SEM).

Parameters	Female	Male	*p*-values	Significance	Reference value [33,36]
Glucose (mg/dl)	66.54 ± 7.26	91.20 ± 12.29	0.088		22.33–97.49
AST (U/l)	9.29 ± 0.67	9.53 ± 0.95	0.843		24.21–93.40
ALT (U/l)	6.17 ± 0.47	5.25 ± 0.34	0.243		7.17–48.48
Creatinine (mg/dl)	2.40 ± 0.19	1.29 ± 0.19	0.002	**	1.07–2.52
Uric Acid (mg/dl)	2.68 ± 0.26	4.22 ± 0.11	0.001	**	
Urea (mg/dl)	47.05 ± 2.06	45.69 ± 2.38	0.704		
Magnesium (mEq/l)	2.12 ± 0.04	2.03 ± 0.06	0.189		2.20–3.93
Phosphorus (mg/dl)	3.62 ± 0.53	3.64 ± 0.79	0.985		5.71–10.35
Calcium (mg/dl)	8.71 ± 0.75	8.30 ± 0.30	0.736		8.72–12.3
Zinc (mg/dl)	118.88 ± 4.39	116.74 ± 5.85	0.785		0.05–0.13
Potassium (mmol/l)	4.96 ± 0.10	5.25 ± 0.12	0.113		3.55–5.9
Sodium (mmol/l)	150.72 ± 6.30	153.17 ± 11.71	0.844		125.7–164.8
Chloride (mmol/l)	102.88 ± 7.80	105.20 ± 4.82	0.854		73.41–116.78

* p < 0.05.

** p < 0.01.

*** p < 0.001.

SEM = Standard error of mean.

## Conclusions

In conclusion, the blood values measured were mostly within reference ranges, which indicates that Murrah buffaloes may acclimatize to saline water with minimum impairment to their liver and renal functions. However, buffaloes with skin lesions showed higher levels of serum AST, creatinine, zinc, sodium, and chloride. It is well established that zinc deficiency has direct links to various skin diseases, but it is unknown whether excessive zinc levels have any toxic effects on the skin. As in this study, sodium is a key element that the buffaloes consistently consume in higher amounts; therefore, finding the actual physiological mechanism requires further investigation to discover the role of sodium in regulating zinc concentrations in the body.

## List of abbreviations

ALT, Alanine aminotransferase; AST, Aspartate aminotransferase; GFR, Glomerular filtration rate; mEq/l, Milliequivalents per liter; ppt, Parts per thousand.
